# Supplementary material for: Cross-Sectional and Longitudinal Associations between Peak Expiratory Flow and Frailty in Older Adults
Source: J Clin Med. 2019 Nov 7;8(11):1901. doi: 10.3390/jcm8111901 (PMC6912606; doi:10.3390/jcm8111901)
Supplement: Supplementary file 1 [file jcm-08-01901-s001.pdf]

SUPPLEMENTARY MATERIAL

Appendix 1. Characteristics of the included and excluded study participants at baseline

Compared to the 2559 individuals included in the cross-sectional analysis, those excluded because of missing data (n=613) were more likely to be older (mean age 82.8±10.6 vs 71.7±9.7 years, $p<0.001$), to be women (73.6% vs. 61.3%, $p<0.001$), and to have lower educational level (15.5% vs. 38% university graduates, $p<0.001$) and cognitive performance (49.8% vs. 12.5% had a Mini-Mental State Examination [MMSE] <28). Moreover, they had worse clinical status (mean number of chronic diseases 5.4±2.9 vs 3.6±2.2, $p<0.001$), with higher prevalence of diagnosed chronic obstructive pulmonary disease (7.7% vs. 4.1%, $p<0.001$) and cardiovascular diseases (41.1% vs. 19%, $p<0.001$).

Similarly, compared to the 2026 individuals included in the longitudinal analysis, those excluded from the cross-sectional sample because of missing data or drop-outs (n=320) were more likely to have lower and cognitive performance (13.8% vs. 9.9% had a MMSE <28, $p=0.04$), but did not show any significant difference on age, sex distribution, number of chronic diseases, prevalence of cardiovascular and of chronic obstructive pulmonary diseases.

Supplementary Table S1. Multivariate regression models and predictive equations for peak expiratory flow from a subsample of healthy SNAC-K participants

	**Men (n=212)**		**Women (n=504)**	
	**Coefficient (SE)**	**Mean (min-max)**	**Coefficient (SE)**	**Mean (min-max)**
Intercept (l/min)	254.58 (193.27)	537.6 (250.0-780.0)	385.78 (97.42)	367.5 (35.0-580.0)
Age (years)	-5.21 (0.71)***	69 (60-96)	-4.93 (0.35)***	73 (60-99)
Height (cm)	3.63 (1.03)***	177 (160-194)	2.11 (0.53)***	162 (142-180)
Predictive equation	254.58 – 5.21*Age + 3.63*Height<br>SDR=88.65, R^2=0.25		385.78 - 4.93*Age + 2.11*Height<br>SDR=73.06, R^2=0.35	

Abbreviations: SE, standard error; SDR, standard deviation of the residuals; R^2, R-squared.
*p<0.05; **p<0.01; ***p<0.001.

Supplementary Table S2. Longitudinal association between peak expiratory flow and frailty over a 6-year follow-up in participants free from physical deficits at baseline (n=1435)

		Odds ratios and 95% Confidence Interval of Frailty	
	**n**	**Model 1**	**Model 2**
**Peak expiratory flow SR-percentile**			
Per each 10th decrease	1435	1.20 (1.10-2.32)***	1.20 (1.09-1.33)***
80th-100th	199	[ref]	[ref]
50th-79th	563	3.02 (1.22-7.50)*	3.06 (1.20-7.78)*
10th-49th	564	4.61 (1.88-11.35)**	4.40 (1.73-11.20)**
<10th	109	6.70 (2.20-20.43)**	7.39 (2.29-23.88)**

Abbreviations: SR, standardized residual. Model 1 is adjusted for age, sex (male vs female), educational level (elementary vs high school vs university), and study time (time to frailty/to follow-up/to death, as appropriate). Model 2 is also adjusted for body mass index, smoking habits (never vs former/current), drinking habits (never/occasionally vs moderate/heavy), baseline Mini-Mental State Examination, number of chronic diseases, chronic obstructive pulmonary disease, asthma, cardiovascular diseases, use of bronchodilators (yes vs no).
*p-value <0.05; **p-value <0.01; ***p-value <0.001.
